# Application of pocket-first technique for implantation of totally implantable venous access ports

**DOI:** 10.1186/s12893-024-02404-4

**Published:** 2024-04-20

**Authors:** Jingjin Wu, Li Zhang, Xiaojian Jia, Yunchuan Mu, Yanbo Lou

**Affiliations:** 1 General Surgery, The Fourth Affiliated Hospital, Zhejiang University School of Medicine, N1 Shangcheng Avenue, Yiwu, China; 2 Nephrology, The Fourth Affiliated Hospital, Zhejiang University School of Medicine, N1 Shangcheng Avenue, Yiwu, China

**Keywords:** TIVAP, Surgery, Pocket, Application

## Abstract

**Background:**

While vascular puncture is always performed before making port pocket in the implantation of totally implantable venous access ports (TIVAP), some surgeons preferred to make port pocket first. This study seeks to verify the safety and feasibility for the pocket-first technique.

**Methods:**

The study retrospectively reviewed 447 patients who undergone TIVAP implantation from July 2017 to November 2022. All the patients were divided into two groups based on vascular puncture first or making port pocket first. The general information, operation information and post-operative complications were reviewed and analyzed.

**Results:**

All the operations were performed successfully. No difference was observed in the sex, age, height, weight, BMI, port location and total complication rate between the two groups. The operation time of the Puncture Group and the Pocket Group were 46.9 ± 22.4 min and 33.8 ± 13.6 min ( *P*<0.00001 ). In the patients of SCV approach, the operation time between the two groups were 37.4 ± 14.8 min and 33.5 ± 10.9 min ( *P*<0.05 ). Multivariate analysis showed the variable BMI and first procedure were independent prognostic factors for operation time. In the cases using SCV/AxV approach the variable first procedure was the only independent prognostic factor for operation time (*P* = 0.002).

**Conclusions:**

The pocket-first technique can be considered as a safe, feasible and convenient technique for TIVAP implantation. The time consuming is significantly shortened compared with the puncture-first technique and this advantage may be more obvious when using SCV/AxV approach.

## Background

Since the initial application of totally implantable central venous access port (TIVAP) in 1982 by Niederhuber Je Fau [1], the technique had spread throughout the world and its safety, effectiveness and convenience had been widely recognized [2, 3]. Wang et al. conducted a meta-analysis to compare the risk of venous thromboembolism (VTE) between peripherally inserted central catheter (PICC) and TIVAP. A lower VTE risk was observed in the TIVAP group regardless of study characteristics, countries and operators [4]. Despite many innovative surgical skills shared from different operators, puncture-first technique was still the mainstream [5], which meant the port pocket was always made after vein puncturing. However, we found that it was not convenient to make incision and separate subcutaneous tissue along the guide wire when it had been inserted. Besides, the operator should pay close attention constantly to protect the guide wire or catheter from pulled out or over inserted. As is well known to all, central vein puncture technique has been more and more mature whether ultrasound-guided (US) or not. Seo et al. reported to perform the TIVAP implantation using a single-incision with high technical success rate and low risk of complications [6]. We further modify the technique to make the port pocket before vein puncture. Based on initial impressions of our work practice, making pocket first seemed to optimize and hasten the implantation without increased complication rate. Consequently, the present study aims to compare the operation time, safety and complications between the puncture-first technique and the pocket-first technique.

## Methods

From July 2017 to November 2022, a list of 447 patients admitted to our hospital and undergone TIVAP implantations was generated by searching our hospital information system. All of the 447 patients were included in the present study. Before January 2021, the puncture-first technique was applied for 278 patients. After January 2021, we used the pocket-first technique for 169 patients. All TIVAPs were from Bard Access Systems, Inc. Pre-operative informed consents were obtained from all patients and the operations were performed by an experienced surgical team. The patients were divided into two groups according to the operative procedures. While the Puncture Group was defined that the patients were undergone vascular puncture before making port pocket, the Pocket Group was defined that the patients were undergone port pocket making before vascular puncture. The general information, operation information and post-operative complications were reviewed. Significant bleeding was defined as hemostasis requiring electro-coagulation or suture. This study was approved by the ethics committee of the Fourth Affiliated Hospital, Zhejiang University School of Medicine (NO: K2023028) and was performed in accordance with the Declaration of Helsinki. Informed consent was waived due to the retrospective nature of the study.

## Patient follow-up

Patients were requested to achieve port maintenance monthly. The X-ray chest radiography was applied when the adverse events were observed such as catheter obstruction. In most cases, the patients received CT scan of the lungs for assessing the tumor growth regularly. Therefore, the CT examination contributed to judge the port morphology and catheter position. Post-operative complications were observed and recorded from the implantation day to the removal day or December 15, 2022.

### Statistical analysis

All data were analyzed using the SPSS 27.0. Continuous variables are presented as‾*x* ± *s* and categorical variables as frequencies (%). For the statistical analysis, a two-sided value of *p* < 0.05 was considered an indication of statistical significance. Patient and operation-related factors that influenced the outcomes were analyzed in univariate and multivariate analysis. Statistical analysis was carried out using the Pearson’s Chi-squared-test or Fisher’s exact test in cross tables, independent sample t-test is used in the comparison of mean values of quantitative information.

### Surgical technique

#### The Pocket Group

**Fig. 1:**
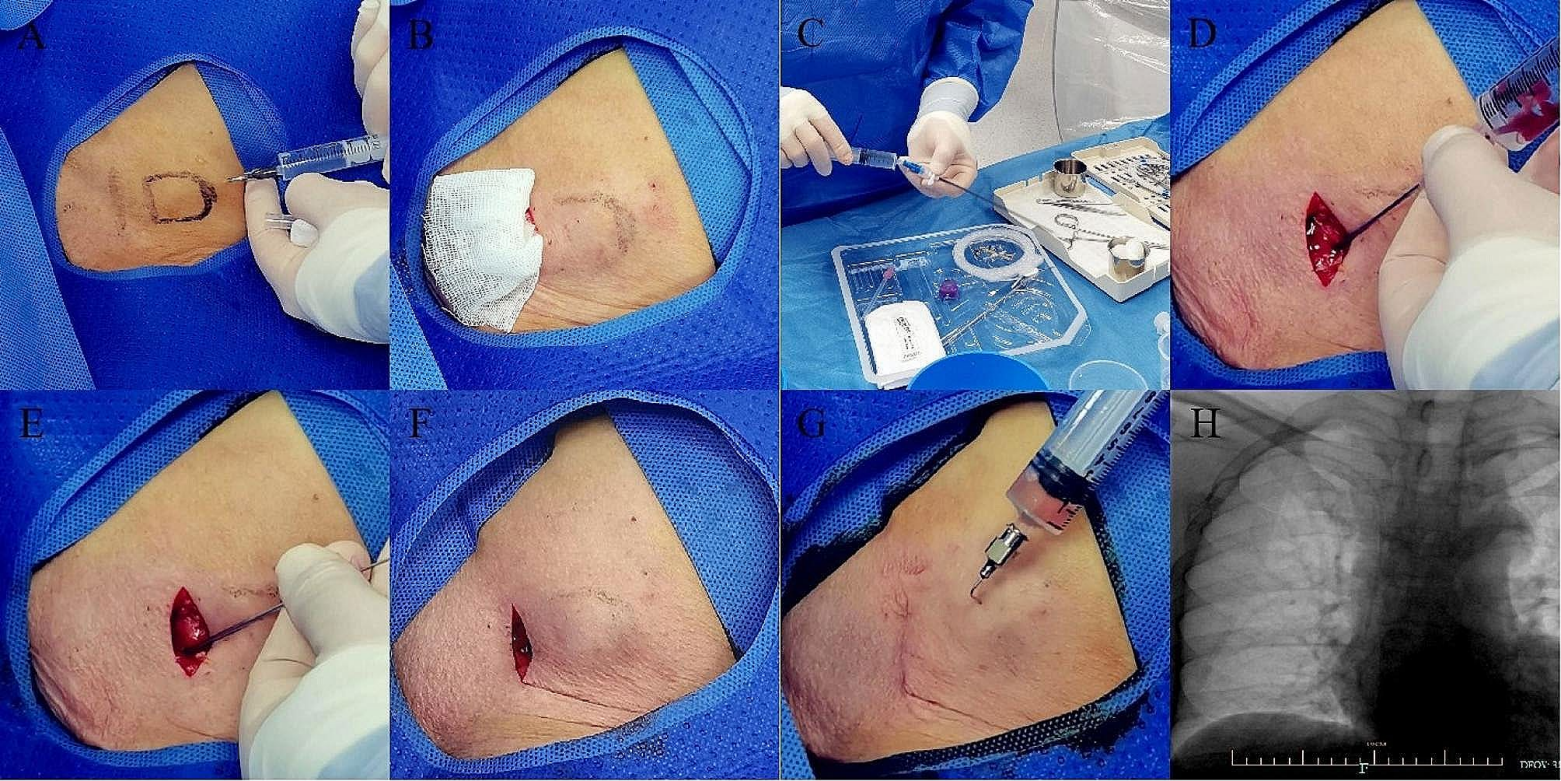
(**A**) Local anaesthesia for making pocket. (**B**) Gauze packing hemostasis. (**C**) Check the integrity of the portal and wash the portal and catheter with heparin saline. (**D**) Puncture of the axillary vein from the bottom plane of pocket. (**E**) Introduce the guide wire. (**F**) The portal is placed into the port pocket after connection of the catheter and portal. (**G**) Suction test with heparin saline to ensure the patency. (**H**) Fluoroscopy for confirming a well-adjusted course and radian of the catheter

1. Local infiltration anesthesia is performed (lidocaine 10 mg/mL + ropivacaine 2.5 mg/mL) (Fig. 1A). A transverse incision (2.5 cm) is made 2 cm below the midpoint of the clavicle and 2 cm inside the deltoid muscle. The port pocket is performed at the below side of the incision by blunt dissection of subcutaneous tissue. Gauze packing hemostasis is used for 5 min (Fig. 1B).

2. During the 5 min above, the surgeon should check the integrity of the portal and wash the portal and catheter with heparin saline (Fig. 1C). Puncture of the axillary vein (AxV) or subclavian vein (SCV) is firstly considered (Fig. 1D). The guide wire is inserted after successful blood extraction and then the peel-away sheath is introduced along the guide wire (Fig. 1E). Next, the catheter is sent to the junction of superior vena cava and right atrium (CAJ) under fluoroscopy. The puncture process can be guided by ultrasound. If three consecutive attempts failed, puncture of the internal jugular vein (IJV) or separation of the cephalic vein should be considered. If the IJV was chosen, then the catheter should be carefully pulled through a subcutaneous tunnel from the puncture point to the port pocket.
3. Cut off the catheter and connect it with the portal, followed with a suction test with heparin saline to ensure the patency.
4. The portal is placed into the port pocket (Fig. 1F) and fluoroscopy is performed again to confirm a well-adjusted course and radian of the catheter.
5. The portal is fixed with fascia using the 4 − 0 non-absorbable suture. Intradermic suture is preferred to close the incision. The last suction test (Fig. 1G) and fluoroscopy (Fig. 1H) are performed at the end.

#### The Puncture Group

1. Puncture of the IJV, SCV or AxV was firstly performed. Whether using the US guidance or not was depended on the surgeon. The guide wire is inserted after successful blood extraction and then the peel-away sheath is introduced along the guide wire. Next, the catheter is sent to the junction of superior vena cava and right atrium (CAJ) under fluoroscopy.
2. Local infiltration anesthesia is performed (lidocaine 10 mg/mL + ropivacaine 2.5 mg/mL). A transverse incision (2.5 cm) is made at the anterior chest wall. The port pocket is performed at the below side of the incision by blunt dissection of subcutaneous tissue. Gauze packing hemostasis is used for 5 min.
3. Then the catheter was carefully pulled through the subcutaneous tunnel, which was made using the tunneling needle from the incision of the port pocket to the puncture point. The scale and radian of the catheter should be adjusted to avoid corner folding.
4. Cut off the catheter and connect it with the portal, followed with a suction test with heparin saline to ensure the patency.
5. The portal is placed into the port pocket and fluoroscopy is performed again to confirm a well-adjusted course and radian of the catheter.
6. The portal is fixed with fascia using the 4 − 0 non-absorbable suture. Intradermic suture is preferred to close the incision. The last suction test and fluoroscopy are performed at the end.

## Results

The clinical characteristics of the patients were listed in the Table 1. The two groups of patients were similar with respect to the sex, age, height, weight, BMI and port location. All the operations were performed successfully. No more than one piece of gauze was wet through blood during each operation which meant intra-operative blood loss was few. The clinical information of surgery and complication was present in the Table 2. All the cases of IJV approach in the two groups and SCV approach in the Puncture Group used US guidance. The operation time of the Puncture Group and the Pocket Group were 46.9 ± 22.4 min and 33.8 ± 13.6 min ( *P*<0.00001 ). In the patients of SCV approach, the operation time between the two groups were 37.4 ± 14.8 min and 33.5 ± 10.9 min ( *P*<0.05 ). In the patients of US guided SCV approach, the operation time between the two groups were 37.4 ± 14.8 min and 33.3 ± 11.0 min ( *P*<0.05 ). The most common complication was catheter malposition (18/447), among which 6, 10 and 2 cases occurred in IJV, SCV and AxV approach respectively. No difference was observed in the total complication rate between the two groups (*P* = 0.189). The variables of patient characteristics and surgery information had no significant influence to the catheter malposition and the total complication rate. The overall follow-up time were 350.6 ± 300.8 days of the Puncture Group and 233.6 ± 196.9 days of the Pocket Group (*P*<0.0001). Univariate analysis revealed that the variables sex, age, height, weight, diagnosis and port location did not correlate with the TIVAP implantation time. Multivariate analysis showed the variable BMI and first procedure were independent prognostic factors for operation time. Significant correlation was seen among the variables first procedure, vein chosen and ultrasonic guidance mutually (Table 3). In the cases using SCV/AxV approach the variable first procedure was the only independent prognostic factor for operation time (*P* = 0.002).

**Table 1:** Clinical Characteristics of patients

		Puncture Group	Pocket Group	*P*
Total		278	169	
Sex				0.33
	Male	136(48.9%)	91(53.8%)	
	Female	142(51.1%)	78(46.2%)	
Age (year)		59.1 ± 11.4	60.6 ± 12.1	0.188
Height (cm)		161.6 ± 7.2	162.2 ± 7.3	0.415
Weight (kg)		59.6 ± 10.4	61.1 ± 9.7	0.141
BMI (kg/m^2^)		22.8 ± 3.3	23.2 ± 3.3	0.179
Diagnosis				<0.0001
	Lung cancer	14(5.0%)	28(16.5%)	
	Breast cancer	73(26.3%)	40(23.7%)	
	Gastric cancer	53(19.1%)	25(14.8%)	
	Pancreatic cancer	9(3.2%)	15(8.9%)	
	Colon cancer	63(22.7%)	23(13.6%)	
	Rectal cancer	41(14.7%)	16(9.5%)	
	Other	25(9.0%)	22(13.0%)	

**Table 2:** Clinical information of surgery and complication

		Puncture Group	Pocket Group	*P*
Port location				0.162
	Left	44(15.8%)	36(21.3%)	
	Right	234(84.2%)	133(78.7%)	
Vein				<0.00001
	Internal jugular vein	186(66.9%)	6(3.6%)	
	Subclavian vein	92(33.1%)	97(57.4%)	
	Axillary vein	0	66(39.0%)	
Ultrasonic guidance				<0.00001
	Yes	278(100%)	113(66.9%)	
	No	0	56(33.1%)	
Operation time (min)		46.9 ± 22.4	33.8 ± 13.6	<0.00001
	IJV approach	51.9 ± 23.8	70.3 ± 22.1	0.063
	SCV approach	37.4 ± 14.8	33.5 ± 10.9	0.045
	AxV approach	NA	30.9 ± 11.6	
	US guided IJV approach	51.9 ± 23.8	70.3 ± 22.1	0.063
	US guided SCV approach	37.4 ± 14.8	33.3 ± 11.0	0.034
	US guided AxV approach	NA	36.8 ± 15.6	
Follow-up time (day)		350.6 ± 300.8	233.6 ± 196.9	<0.0001
Complication		17(6.1%)	16(9.5%)	0.189
	Inadvertent arterial puncture	0	3(1.8%)	
	Pneumothorax	1(0.4%)	1(0.6%)	
	Pain	1(0.4%)	1(0.6%)	
	Catheter-Related Blood Stream Infection	0	1(0.6%)	
	Catheter associated thrombosis	1(0.4%)	1(0.6%)	
	Skin flap rupture	1(0.4%)	1(0.6%)	
	Catheter occlusion	1(0.4%)	2(1.2%)	
	Catheter malposition.	12(4.3%)	6(3.6%)	

**Table 3:** Predicting factors of TIVAP operation time

Univariate analysis				Correlation analysis				Multivariate analysis		
	*R²*	ANOVA *P*			Pearson’s correlation	*P*			*R²*	*P*
First procedure	**0.099**	**<0.001**		First procedure vs. BMI	0.064	0.179		total	0.111	**<0.001**
Sex	0.00049	0.642		First procedure vs. Vein	0.704	**<0.001**		First procedure		**<0.001**
Age	0.0038	0.194		BMI vs. Vein	0.009	0.867		BMI		**0.014**
Height	0.000043	0.89		First procedure vs. Ultrasonic guidance	0.912	**<0.001**				
Weight	0.0055	0.117								
BMI	**0.0082**	**0.056**								
Diagnosis	0.00045	0.653		Vein vs. Ultrasonic guidance	0.786	**<0.001**				
Port location	0.000059	0.872								
Vein	**0.179**	**<0.001**								
Ultrasonic guidance	**0.102**	**<0.001**		BMI vs. Ultrasonic guidance	0.050	0.292				

## Discussion

As the TIVAP has been widely used due to its safety and convenience, the options of venous approach and port location are various. The IJV approach is commonly used because of its advantages such as wide diameter and shallow position. However, two incisions and a subcutaneous tunnel are required, which may bring about some discomfort due to the catheter in the subcutaneous tunnel and influence the cosmetic appearance. A randomized controlled study had been launched to compare of comfort and complications after port implantation between ultrasound-guided IJV approach and AxV/SCV approach [7]. Significant higher comfort level was observed in the AxV/SCV group. None discomfort (grade 0) appeared in 66.9% patients of the AxV/SCV group at day 1 [8]. The foreign body sensation in neck was the most obvious discomfort, which also influenced their neck movement.

Up to now, the optimal central venous insertion remains controversies. Although the IJV insertion is seemingly more widely used, the AxV/SCV insertion is not uncommon. J L Westcott reported the first percutaneous axillary vein approach in 1972 [9]. Nickalls et al. reported a percutaneous landmark access to the axillary vein [10]. Furthermore, the X-ray fluoroscopy, phlebography and US-guide technique were also applied to the puncture of axillary vein [11–13]. Wu et al. analyzed 3905 patients to compare the complication rate between the IJV and SCV approaches for TIVAP implantation. The incidence of TIVAD-related infections, catheter fracture and catheter-related thrombotic complications were not significantly different between the two groups. However, the IJV group had a lower risk of total major mechanical complications (OR 0.38, 95% CI 0.24–0.61, *P* < 0.001) and the SCV group appeared more prevalent of catheter dislocation (OR 0.43, 95% CI 0.22–0.84, *P* = 0.013) and malfunction (OR 0.42, 95% CI 0.28–0.62, *P* < 0.001) [14]. A meta-analysis including 1086 patients conducted by Zhou et al. showed little difference in the complication rate of TIVAP between IJV and SCV insertion [15]. A prospective randomized study conducted by Han L et al. concluded that SCV should be the first choice for TIVAP implantation in children. In the study, the SCV group had a comparative advantage in the postoperative catheter occlusion rate (3 vs. 10, *P* < 0.05), operation time (45.7 min vs. 75.9 min, *P* < 0.001) and satisfaction score (9.6 vs. 8.3, *P* < 0.001) [16].

In our initial practice, the IJV approach was applied for port implantation. After sufficient literature review and further study, we started to use a percutaneous landmark access to the SCV. However, making an incision along the guide wire or catheter is inconvenient and the surgeon must pay attention to protect the guide wire or catheter when making the port pocket. Some studies have reported only a single incision was needed by the AxV/SCV approach with good security and feasibility [6, 17]. Therefore, we preferred to puncture the SCV through the infraclavicular incision. Then we found corner folding was easily observed in the junction of the port and catheter because the puncture point was always higher than the bottom plane of pocket. As a result, we began to make the pocket firstly and puncture the SCV from the bottom plane of pocket. Lately the AxV approach was also used in the port implantation. That is why the follow-up time in the Puncture Group was significantly longer than the Pocket Group.

In the present study, the success rate of implantation was 100% and no statistical significance was observed in the complication rate between the two groups. There were three cases of accidental arterial puncture during operation. However, it was interesting that the ultrasound-guided technique was used in two cases, which we considered may be related to the lack of experience in the combination of the pocket-first technique and the ultrasound-guided technique in the early cases. Catheter malposition was the most common complication in the present study and the occurrence rate in SCV approach (10/189, 5.3%) was higher than IJV (6/192, 3.1%) and AxV (2/66, 3.0%). Some studies had shown that the catheter malposition rate using SCV approach was higher than IJV [14, 18, 19]. However, there was lack of adequate statistical power for the correlation between the vein approach and the catheter malposition in our study.

The operation time in the Pocket Group was shorter compared with the Puncture Group. The mean operation time was 33.8 ± 13.6 min in the Pocket Group, which was shorted than other studies [16, 20]. Univariate analysis revealed that the variables first procedure, vein chosen, ultrasonic-guidance and BMI were correlated with the TIVAP implantation time. Multivariate analysis showed the variable BMI and first procedure were independent prognostic factors. Significant correlation was seen among the variables first procedure, vein chosen and ultrasonic guidance mutually. This statistical result may be on account of the distribution of cases. For example, the cases using the IJV approach were almost in the Puncture Group and the AxV were all in the Pocket Group. However, it’s worth noting that the case number using SCV approach between the Puncture Group and the Pocket Group seemed similar. The operation time was shorter in the Pocket Group when using the SCV approach no matter utilizing the ultrasound-guided technique or not. Besides, the variable first procedure was the only independent prognostic factor for implantation time using SCV/AxV approach. Therefore, there is every reason to believe of less time consuming when using the pocket-first technique especially in patients using the SCV/AxV approach.

It is well known that venipuncture and pocket making are core steps for both of the TIVAP implantation and cardiac implantable electronic device (CIED) implantation. To the best of our knowledge, there is little literature report on the use of the pocket-first technique in CIED implantation. Many studies had shown that the ultrasonic-guided AxV access for CIED is equally feasible, safe, and faster than the IJV, SCV and cephalic vein access [21–23]. In our study, the operation time using AxV access was the shortest. Therefore, AxV seems the ideal access for central venous puncture and catheterization. It is a pity that there no patient using the AxV approach included in the Puncture Group. Further researches should be conducted to validate the advantages of the pocket-first technique in TIVAP implantation via the AxV approach.

The time interval between TIVAD placement and the first use of the TIVAD is still a controversy. Many studies suggested the interval of at least 24 h was safe [24, 25]. However, Ozdemir et al. considered that the first chemotherapy immediately after implantation did not increase the acute or chronic complications [26]. Karanlik et al. investigated 1315 patients who were divided into two groups according to whether chemotherapy was administered within 24 h. The frequency of early and late complications did not differ statistically significantly between the two groups [27]. There was a survey conducted to determine the knowledge levels of oncology nurses about peripheral and central venous catheter. 67.3% of the 165 nurses responded correctly to the time interval between the insertion of port catheter and the first chemotherapy, which meant the first chemotherapy should be done as soon as possible after port insertion [28]. In a study for predictors of port-related venous thrombus, chemotherapy within 0 to 8 days after port placement was not associated statistically with 3-month catheter-related VTE event [29]. Therefore, all the patients started first chemotherapy within 24 h in our study.

Catheter tip position plays an important role in a port implantation. As is well known, the cavoatrial junction (CAJ) is regarded as the ideal position. Nevertheless, the CAJ is difficult to confirm in the real world. Intraoperative fluoroscopy, intracardiac electrocardiogram technology and postoperative chest radiograph are the main three methods to confirm the position of the catheter tip [30]. Caers et al. reviewed 437 patients to identify predisposing factors of different complications. The incidence of thrombosis was 8.46% which was the most common late complication. Five categories were defined for reviewing the positioning of the catheter tip by chest radiographs or phlebographies. The minimum incidence of thrombosis was 1.5% (*P* < 0.001) when the catheter tip located at category IV which meant the caudal third of the superior vena cava (SVC) [31]. Therefore, the ideal catheter tip position of the lower third of the SVC is recommended in many guidelines [3, 32, 33]. As no intracardiac electrocardiogram monitoring can be used, intraoperative fluoroscopy or postoperative chest radiograph was applied for confirming the catheter tip position in the present study.

Several limitations exist in the present study. First, it was a retrospective study of one single center with a limited sample size and multicenter randomized controlled trials are required. Second, most patients chose to extract the port at the end of 3–6 months treatment. Long-term follow-up is also needed. Third, the factors related to satisfaction and aesthetics should be included because many patients prefer TIVAP for its comfort and beauty.

## Conclusions

The pocket-first technique can be considered as a safe, feasible and convenient technique for TIVAP implantation. The time consuming is significantly shortened compared with the puncture-first technique and this advantage may be more obvious when using SCV/AxV approach. More researches are required for popularization.

## Data Availability

The datasets used during the current study are available from the corresponding author on a reasonable request.

## Abbreviations

TIVAP: Totally implantable central venous access port
VTE: Venous thromboembolism
PICC: Peripherally inserted central catheter
US: Ultrasound
AxV: Axillary vein
SCV: Subclavian vein
CAJ: Junction of superior vena cava and right atrium
IJV: Internal jugular vein
SVC: Superior vena cava
CIED: Cardiac implantable electronic device
